# The Flot2 component of the lipid raft changes localization during neural differentiation of P19C6 cells

**DOI:** 10.1186/s12860-019-0225-0

**Published:** 2019-08-27

**Authors:** Kei Hanafusa, Nobuhiro Hayashi

**Affiliations:** School of Life Science and Technology, Tokyo Institute of Technology, Meguro-ku, Tokyo, Japan

**Keywords:** Lipid raft, Neural differentiation, Flot2, Fyn, C-Src, P19C6 cells

## Abstract

**Background:**

Flotillin-2 (Flot2) is a lipid raft scaffold protein that is thought to be related to neural differentiation. Flot2 is phosphorylated by Fyn, a Src kinase, and causes raft-dependent endocytosis; however, the exact role of Flot2 in neural differentiation remains unclear. To reveal the roles of lipid raft-associated proteins during neural differentiation, we tried to analyze the expression and localization.

**Results:**

In this study, we found that the expression levels of the Flot2 and Fyn proteins increased in whole-cell lysates of P19C6 cells after neural differentiation. In addition, sucrose density fractionation and immunofluorescence experiments revealed an increase in the localization of Flot2 and Fyn to lipid rafts after neural differentiation. We also found that Fyn partially colocalized with Flot2 lipid rafts in neural cells.

**Conclusion:**

The observed distribution of Fyn and level of inactivated Fyn and/or c-Src in detergent–resistant membrane (DRM) fractions suggests that the amount of activated Fyn might increase in DRM fractions after neural differentiation. Overall these findings suggest that Flot2 lipid rafts are associated with Fyn, and that Fyn phosphorylates Flot2 during neural differentiation of P19C6 cells.

**Electronic supplementary material:**

The online version of this article (10.1186/s12860-019-0225-0) contains supplementary material, which is available to authorized users.

## Background

Neural differentiation is a remarkable phenomenon that contributes to the formation and function of the central nervous system. The differentiation process makes use of lipid rafts, which are microdomains present on the plasma membrane that contribute to signal transduction, cell-cell adhesion, neurite elongation, and the function of receptors for neurotrophic factors [1–7]. Therefore, analyses of lipid rafts are expected to yield important information for understanding the development and function of the central nervous system.

Lipid rafts are formed in specific regions that are abundant in glycosphingolipids and cholesterol; these regions can be isolated by sucrose density fractionation as a low buoyant density fraction [8–10]. Lipid rafts promote intracellular signaling by associating with acylated proteins [7, 11–13].

The flotillin protein family contains two members, flotillin-1 (Flot1) and flotillin-2 (Flot2), which are expressed constitutively in cells, highly homologous, and conserved from bacteria to mammals [7, 14–16]. Flots were initially identified as gene products that were upregulated in optic nerve lesions and during axon regeneration in goldfish [17]. Subsequently, Flots were found to be essential for neurite elongation, and *Flot2* knockout mice exhibit impaired neuron maturation [18], hence, Flot2 is thought to play a role in neural differentiation.

Flot1 and Flot2 are localized to lipid raft regions via palmitoylation (Flot1 and Flot2) and myristoylation (Flot2). These proteins act as scaffolds for lipid rafts by forming homo- and hetero-oligomers [19, 20]. Flots reportedly interact with cytoskeletal proteins such as tubulin and actin via their stomatin/Prohibitin/Flotillin/HflK/C (SPFH) domains [21, 22]. In particular, tyrosine phosphorylation of Flots by the Src kinases results in the migration of Flots from the vicinity of the cell membrane to the cytosol [23, 24]. Flot2 interacts with *N*-methyl-d-aspartate receptors and interacts with Rab11a-expressing vesicles to promote endocytic transport of postsynaptic density-95, N-cadherin, and glutamate receptors [19, 25, 26].

The tyrosine kinases pp59^Fyn^ (Fyn) and pp60^c-Src^ (c-Src) are lipid raft markers [23] that serve as signal transduction factors for cytoskeletal regulation [22, 27]. Fyn is related to neuronal differentiation [28] and neurite outgrowth is inhibited in *fyn*-minus mice [29]. Fyn and c-Src are localized in lipid rafts as a result of myristoylation and palmytoylation. Ca^2+^-bound calmodulin interacts with myristoylated Fyn and c-Src and regulates their localization [30, 31]. C-terminal Src kinase regulates the phosphorylation of Fyn at Y528 and c-Src at Y530, and these phosphorylated forms are inactive [32, 33]. The inactive forms of Fyn (pY528) and c-Src (pY530) are activated by dephosphorylation [34]. Notably, Fyn and c-Src are bound to membrane-bound γ-tubulin via their SH2 domain and the resulting complexes have been implicated in neural differentiation [35].

Neural differentiation can be modeled using P19C6 cells, which are subclones of a multipotent embryonic carcinoma cell line P19. Stimulation of P19 cells with high concentrations of retinoic acid results in the formation of embryonic bodies and induces neural differentiation. To our knowledge, studies of lipid rafts in P19 cells have not been performed to date. Moreover, P19 would not express Flot1, and the expression of Flot2 in this cell line has been reported [16]. Therefore, in the present study, we examined the functions of lipid rafts during neural differentiation in the P19C6 cell line by examining the localizations of Flot2, Fyn, and c-Src as lipid raft components.

## Results

### Flot2 expression during neural differentiation of P19C6 cells

**Fig. 1:**
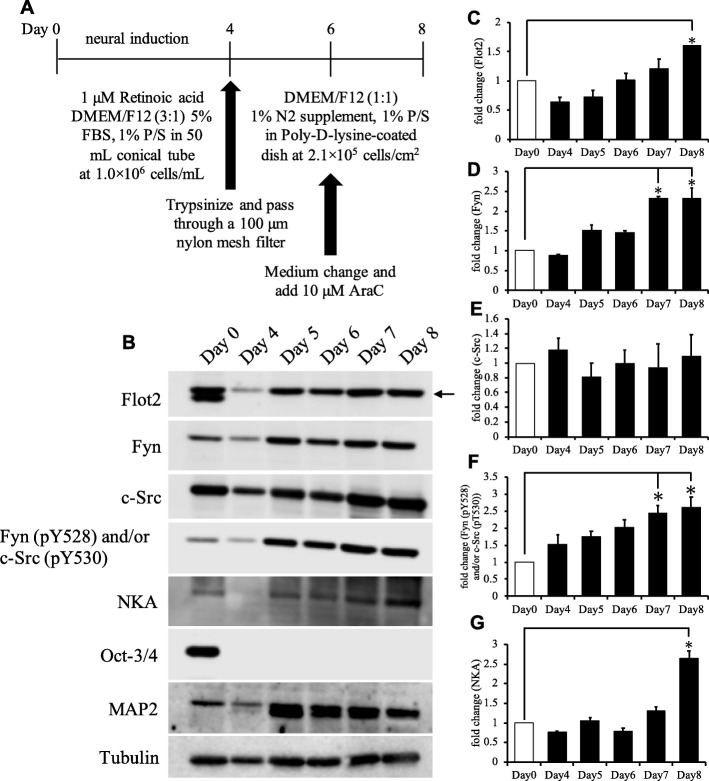
Protein expression levels differ between undifferentiated cells (Day 0) and neural differentiated cells (Day 8). **a** Schematic overview of the method used to induce neural differentiation of P19C6 cells. **b** Western blotting analyses of whole-cell lysates at Days 0 and 4–8 after neural induction. Cropped images are shown. The blots were probed with an anti-Flot2 antibody (arrow), anti-Fyn antibody, anti-c-Src antibody, anti-Fyn (pY528) and/or c-Src (pY530) antibody, anti-Na/K ATPase α1 subunit antibody (NKA), anti-Oct-3/4 antibody as a stem cell marker, anti-MAP2 antibody as a neural cell marker, and anti-tubulin antibody as a loading control. **c**-**g** Densitometric band intensities of Flot2 (**c**), Fyn (**d**), c-Src (**e**), Fyn (pY528) and/or c-Src (pY530) (**f**), and NKA (**g**) on Days 0 and 4–8. The band intensities were normalized to that of tubulin. Data represent the mean ± SEM (*n* = 4); *p < 0.05 by an unpaired Student’s t-test. Abbreviations: AraC: Cytosine β-D-arabinoside; MAP2: Microtubule-associated protein 2; Flot2: Flotillin2; NKA: Na/K ATPase

The expression level of the mRNA encoding Flot2 changes during neural differentiation of human multipotent stromal cells (hMSCs) [36]. To determine whether the expression level of the Flot2 protein also changes during neural differentiation, we used western blotting (WB) to compare Flot2 protein levels in undifferentiated P19C6 cells with those undergoing neural differentiation (Fig. 1a). The level of Flot2 in whole-cell lysates was increased with neural differentiation (Fig. 1b). Octamer transcription factor-3 (Oct-3), a stem cell marker protein [37], was detected only on Day 0 (prior to neural induction) (Fig. 1b). On the other hand, the expression level of the neural marker microtubule-associated protein 2 (MAP2) [38] increased from Day 4 to Day 8 of neural induction (Fig. 1b). The expression level of Flot2 increased gradually during neural differentiation and was 1.6-fold higher on Day 8 than on Day 0 (*p* < 0.05; Fig. 1c). Fluorescent microscopy confirmed the presence of morphological changes typical of neural differentiation, including elongated neurites extending from the soma, in the induced cells (Additional file 2: Figure S2).

Next, the levels of Fyn and c-Src, and the combined level of phosphorylated Fyn (pY528) and/or phosphorylated c-Src (pY530), were assessed by WB [39–41]. The expression level of Fyn was 2.3-fold higher on Days 7 and 8 than on Day 0 (*p* < 0.05; Fig. 1d). By contrast, c-Src expression did not change during neural differentiation (Fig. 1e). The combined level of Fyn (pY528) and/or c-Src (pY530) increased gradually during neural differentiation and was 2.7-fold higher on Day 8 than on Day 0 (*p* < 0.05; Fig. 1f).

Na/K ATPase (NKA) is a non-raft and plasma membrane marker that interacts with c-Src and protein kinase C [42, 43]. We used WB to examine the expression levels of the α1 subunit of NKA in differentiated and undifferentiated P19C6 cells. The expression level of NKA on Day 8 was 2.6-fold higher than that on Day 0 (*p* < 0.05; Fig. 1g). This observation is consistent with a previous study showing that NKA expression was 2.64-fold higher on Day 8 than on Day 0 of P19 cell neural differentiation [44].

Based on the results described above, we defined Day 0 as undifferentiated cells (UD) and Day 8 as neural differentiated cells (Neu).

### Localization of lipid raft markers and a non-raft marker during neural differentiation of P19C6 cells

**Fig. 2:**
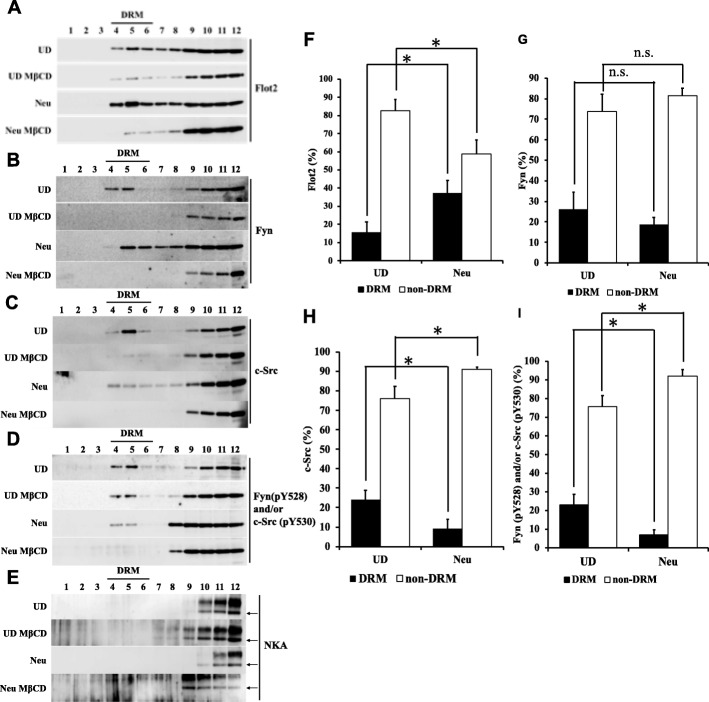
Western blotting analyses of sucrose density fractions. **a**-**e** Representative results of western blotting analyses of sucrose density fractions of undifferentiated (UD) and neural differentiated (Neu) P19C6 cells, treated with or without MβCD. The fractions were incubated with antibodies against Flot2 (**a**), Fyn (**b**), c-Src (**c**), Fyn (pY528) and/or c-Src (pY530) (**d**), and NKA (**e**). Cropped images are shown. **f**-**i** Densitometric intensity analyses of the bands shown in **a**–**d**, representing the relative levels of Flot2 (**f**), Fyn (**g**), c-Src (**h**), and Fyn (pY528) and/or c-Src (pY530) (I) in the DRM (Frs. 4–6) and non-DRM fractions (Frs. 7–12) of UD and Neu cells. Data are represented as the mean ± SEM (*n* = 6); *p < 0.05 by a two-tailed unpaired Student’s *t*-test. Abbreviations: Flot2: Flotillin2; NKA: Na/K ATPase; DRM: Detergent-resistant membrane; MβCD: methyl-β-cyclodextrin

Next, we performed WB analyses of sucrose density fractions to examine the distributions of lipid raft markers (Flot2, Fyn, c-Src, Fyn (pY528) and/or c-Src (pY530)) and a non-raft marker (NKA) before and after neural differentiation of P19C6 cells (Fig. 2a-e). It is known that lipid rafts are present in detergent-resistant membrane (DRM) fractions (Frs) that appear at the interface between sucrose concentrations of 30 and 5%. The levels of fractionated proteins that are associated with lipid rafts are decreased by methyl-β-cyclodextrin (MβCD) treatment, which depletes cholesterol from plasma membrane [45]. MβCD treatment of P19C6 cells reduced the intensities of the bands representing Flot2, Fyn, c-Src, and Fyn (pY528) and/or c-Src (pY530) in Frs 4, 5, and 6 (Fig. 2a-d). NKA was detected in Frs 9 to 12 but not Frs 4, 5, and 6 (Fig. 2e). Therefore, we defined Frs 4, 5, and 6 as DRM Frs, and the other Frs as non-DRM Frs. As shown in Fig. 2a and f, DRM-associated Flot2 was increased after neural differentiation.

In whole-cell lysates, the level of Fyn and the combined level of phosphorylated Fyn (pY528) and/or phosphorylated c-Src (pY530) increased during neural differentiation (Fig. 1d and f). Figure 2 shows the distributions of Fyn, c-Src, and phosphorylated Fyn (pY528) and/or phosphorylated c-Src (pY530) in sucrose density Frs and their recovery ratios in DRM Frs. The recovery ratio of Fyn in DRM Frs did not change during neural differentiation (Fig. 2g). The recovery ratio of phosphorylated Fyn (pY528) and/or phosphorylated c-Src (pY530) in DRM Frs was decreased in Neu, while that in non-DRM Frs was increased (Fig. 2i). By contrast, the level of c-Src did not change during neural differentiation (Fig. 1e), and the recovery ratio of c-Src in DRM Frs was decreased in Neu (Fig. 1h).

Because Flot2 in DRM Frs was increased after neural differentiation, we examined the localization of Flot2 to lipid rafts in UD and Neu P19C6 cells. The cells were co-stained with an anti-Flot2 antibody and Cholera toxin subunit B (CTB), a non-toxic component of cholera holotoxin (Additional file 1: Figure S1A and Additional file 2: Figure S2A). CTB can bind to GM1, a glycosphingolipid containing sialic acid, and forms lipid rafts by its physiochemical property. Flot2 is localized in GM1-containing lipid rafts in T cells [46]. In UD cells, Flot2 seemed to be located proximal to the plasma membrane. Following neural differentiation, Flot2 was located in the neurites and soma (Additional file 2: Figure S2A). In Neu cells, it seems that Flot2 and CTB colocalized signals, which were not colocalized with NKA, were in neurite (Additional file 2: Figure S2A and D).

**Fig. 3:**
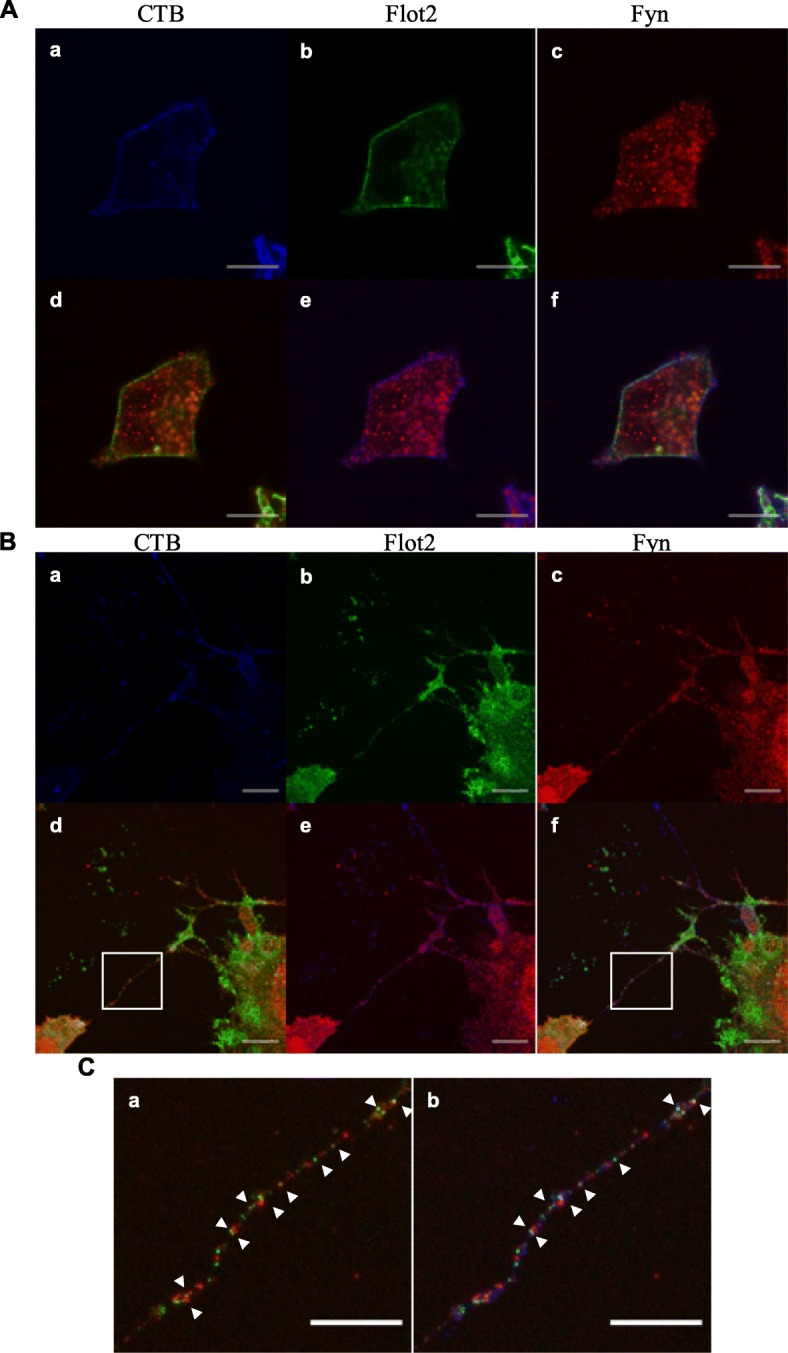
Localization of Flot2 and Fyn before and after neural differentiation. **a**, **b** Undifferentiated (**a**) and neural differentiated (**b**) P19C6 cells were fixed and stained with an anti-Flot2 antibody (green, b), Alexa488-conjugated Cholera toxin subunit B (blue, a), and an anti-Fyn antibody (red, c). Merged images of Flot2 and Fyn (**d**), CTB and Fyn (**e**), and Flot2, Fyn, and Cholera toxin subunit B (**f**) are shown. Scale bar shows 20 μm. **c** Higher magnification images of the white boxes in B-d and B-f. The arrowheads indicate colocalized signals Flot2 and Fyn (C-a) and Flot2, Fyn and CTB (C-b). Scale bar shows 5 μm. Abbreviations: Flot2: Flotillin2; CTB: Cholera toxin subunit B

Next, we compared the localization of Flot2 with that of the lipid raft markers Fyn and c-Src in UD and Neu cells. In both cell types, Fyn showed a spotty distribution whereas c-Src was localized along the cell membrane (Fig. 3, Additional file 1: Figure S1, and Additional file 2: Figure S2). Fyn and c-Src may partially colocalize with Flot2 in UD cells. By contrast, Fyn may partially colocalize with CTB or Flot2 in neurites (Fig. 3c).

## Discussion

To our knowledge, the work presented here is the first lipid raft analysis of P19C6 cells before and after neural differentiation. P19C6 embryonic carcinoma cells are widely used as a neuronal differentiation model. Notably, P19 cells are thought to be more sensitive to chemicals than other in vitro neuronal models, including rat adrenal pheochromocytoma PC12 cells and human neuroblastoma SH-SY5Y cells [47]. Based on this characteristic, we hypothesized that P19 cells could serve as a model for lipid raft analysis of neuronal differentiation induced by retinoic acid.

In P19C6 cells, the Flot2 protein level in whole-cell lysates and DRM Frs and the recovery ratio of Flot2 in DRM Frs were significantly higher than in non-DRM Frs after neural differentiation. In a previous study, neural differentiation of hMSCs induced upregulation of the *Flot2* mRNA level and relocalization of the Flot2 protein to the plasma membrane and lipid raft Frs [36] . However, in PC12 cells, which are frequently used as models of nerve-like cells, Flot1, but not Flot2, is upregulated after neural differentiation [48]. P19 cells and hMSCs are multipotent cells that are able to differentiate into multiple cell types, whereas PC12 cells are derived from the adrenal gland and yield only neuron-like cells upon differentiation [36, 49, 50]. Moreover, P19 and PC12 cells exhibit specific differences in differentiation. Notably, P19 cells resemble the original cells that they were derived from in the embryo, and can differentiate into neural cells by forming embryoid bodies upon retinoic acid stimulation [51].

NKA is a multi-subunit protein [52]. The α1 subunit of NKA is a non-raft and plasma membrane marker that is expressed constitutively in a wide variety of cell types, and NKA activity is related to that of c-Src [53, 54]. NKA interacts with caveolin-1, and the resulting complex forms caveolae-like membrane microdomains that are related to Ca^2+^ signaling via phosphoinotiside-3 kinase [55, 56]. In the current study, NKA was localized at the plasma membrane and was not recovered in the DRM Frs of P19C6 cells. This finding may be explained by the fact that neuronal cells are devoid of caveolin-1 and caveolae [14, 57].

The levels of Flot2 and Fyn increased during neural differentiation of P19C6 cells (Fig. 1c and d). The recovery ratio of DRM-associated Fyn did not change during neural differentiation (Fig. 2b and g); however, the recovery ratio of inactive Fyn and/or c-Src was decreased in DRM Frs (Fig. 2d and i). Therefore, the level of DRM-associated active Fyn might be increased, but the combined level of DRM-associated phosphorylated Fyn (pY528) and/or phosphorylated c-Src (pY530) might be decreased in Neu. These results suggest that level of the active form of Fyn might be increased in DRM Frs during neural cells [58]. Taken together, these results raise the possibility that Flot2 lipid rafts are associated with Fyn, and that Fyn may phosphorylate Flot2 during neural differentiation of P19C6 cells.

The active form of Fyn binds to membrane-bound γ-tubulin via phosphorylated SH2 domains and forms the core of the microtubule organizing center with γ-tubulin during neural differentiation [35]. This core promotes microtubule nucleation by phosphoinositide 3-kinase [28]. Flot2 phosphorylation causes endocytosis, resulting in cargo formation to deliver the endocytosed materials to growth cone outgrowths [25, 59]. Moreover, Flot2 interacts with cytoskeletal proteins in lipid rafts [24]. These observations raise the possibility that Flot2-associated lipid rafts may be involved in morphological changes such as cytoskeletal remodeling during neural differentiation. Further studies are needed to elucidate the roles of Flot2- and Fyn-associated lipid rafts in neural differentiation and neurite outgrowth.

## Conclusion

During neural differentiation of P19 cells, Flot2 tend to localize in DRM Frs. Moreover, our data suggested that not only the observed distribution of Fyn but also the amount of activated Fyn in DRM Frs might be increased during neural differentiation. These findings suggested that Flot2 lipid rafts are associated with Fyn. This association might cause the phosphorylation of Flot2 during neural differentiation. Our findings, the association between lipid raft scaffold protein, Flot2, and acylated proteins such as Fyn, c-Src, may be the key concept of development and function of central nervous system.

## Methods

### Reagents

P19C6 cells were obtained from the RIKEN Bioresource Center Cell Bank (Ibaraki, Japan). Alexa405-conjugated goat polyclonal anti-mouse IgG H&L (No. ab175660; used at a 1:500 dilution) and the rabbit monoclonal anti-Fyn antibody [EPR5500] (ab125016; WB: 1:1000; IF: 1:500) were purchased from Abcam (Cambridge, UK). The mouse monoclonal anti-Flot2 antibody (No. 610383; WB: 1:4000; immunofluorescence: 1:500) and mouse monoclonal anti-Fyn (pY528)/c-Src (pY530) antibody (No. 612668; WB: 1:2000) were purchased from BD Biosciences (Franklin Lakes, NJ, USA). The rabbit monoclonal anti-Src (36D10) antibody (#2109; WB: 1:1000; IF: 1:100) was purchased from Cell Signaling Technology (Danvers, MA, USA). The 2D-Quant kit was purchased from GE Healthcare (Little Chalfont, UK). Horseradish peroxidase (HRP)-conjugated goat polyclonal anti-mouse IgG (No. 330; 1:5000) and HRP-conjugated goat polyclonal anti-rabbit IgG (No. 458; 1:5000) were purchased from MBL (Nagoya, Japan). The Immobilon-P membrane (IPVH00010) and Immobilon Western Chemiluminescent HRP substrate (No. P36599) were purchased from Millipore (Billerica, MA, USA). The mouse monoclonal anti-Oct-3/4 antibody (sc-5279; WB: 1:1000) was purchased from Santa Cruz Biotechnology (Dallas, TX, USA). The mouse monoclonal anti-MAP2 Clone HM-2 antibody (No. M 9942; 1:1000), the Mammalian Cell Lysis Kit, and Cy3-conjugated sheep polyclonal anti-rabbit IgG (No. C2306; 1:500) were purchased from Sigma-Aldrich (St. Louis, MO, USA). Rabbit polyclonal antibodies raised against human α-tubulin (WB: 1:8000) and against human NKA α1 subunit (WB: 1:1000 or IF: 1:100) were produced by Thermo Fisher Scientific (Waltham, MA, USA). Cholera toxin Subunit B (Recombinant), Alexa Fluor 488 conjugate (C22841) was purchased from Thermo Fisher Scientific (Waltham, MA, USA).

### Cell culture

P19C6 mouse embryonic carcinoma cells were grown in high-glucose Dulbecco’s Modified Eagle Medium (DMEM; Thermo Fisher Scientific) supplemented with 10% heat-inactivated fetal bovine serum (FBS; GE Healthcare) and 1% penicillin-streptomycin (P/S; Gibco, Waltham, MA, USA) at 37 °C in at atmosphere containing 5% CO_2_.

### Neuronal differentiation

P19 cells are able to differentiate into neuronal cells after all-*trans*-retinoic acid stimulation and embryoid body formation, as described previously [60]. In the present work, we used a modified version of this method. Briefly, cultured cells were washed three times with phosphate-buffered saline (PBS) and then separated by the addition of 0.25% trypsin containing 1 mM EDTA. The number of cells was counted using standard methods. An aliquot of 1 × 10^6^ cells was cultured in suspension in a 50 mL conical tube containing 20 mL of a 1:1 mixture of DMEM and DMEM/F12 supplemented with 5% FBS, 1% P/S, and 1 μM all-*trans*-retinoic acid (Sigma), in a 5% CO_2_ incubator at 37 °C. After 5 days, the medium was aspirated, leaving the cells that had formed aggregates in the 50 mL conical tube. To dissociate the aggregates, the cells were treated with 0.25% Trypsin and 1 mM EDTA, and triturated with a pipette. DMEM supplemented with 10% FBS and 1% P/S was then added, and the suspension was centrifuged. The supernatant was discarded and cell pellets were resuspended in DMEM supplemented with 10% FBS and 1% P/S. To ensure separation of any remaining aggregates into single cells, cell suspensions were passed through a 100 μm nylon mesh filter (Becton Dickinson, Franklin Lakes, NJ, USA), and the cells were then seeded onto poly-d-lysine-coated dishes at a density of 2.0 × 10^5^ cells/cm^2^.

### Sucrose density gradient fractionation

The culture medium was removed from cell cultures and the cells were washed twice with PBS. Subsequently, the cells were resuspended in lysis buffer (10 mM Tris-HCl (pH 7.4), 1 mM EDTA, 1 mM EGTA, and 1% (w/v) Triton X-100) supplemented with 1 mM Na_3_VO_4_ and 1% protease inhibitor cocktail. After the lysates were placed on ice and homogenized with a Dounce homogenizer, the total volume was adjusted to 2 mL by the addition of lysis buffer. A 2 mL aliquot of 80% sucrose was added to the lysate, and the combination was mixed completely and transferred to a high-speed centrifugation tube. The mixture was then overlaid sequentially with 4 mL of 30% sucrose and 4 mL of 5% sucrose, and the tube was centrifuged at 37,679×*g* for 16 h at 4 °C in a SW41Ti rotor (Beckman, Brea, CA, USA). Twelve fractions were collected starting from the top of the surface of the centrifuged liquid.

### Western blotting

The cells were suspended in Mammalian Cell Lysis Kit buffer containing 1 mM Na_3_VO_4_ and 1% protease inhibitor cocktail, and placed on ice for 30 min to lyse. The protein concentration of the resulting lysate was determined using the 2D-Quant kit. Aliquots of the samples corresponding to 10 μg of total protein were separated by 10% sodium dodecyl sulfate-polyacrylamide gel electrophoresis and transferred to a polyvinylidene fluoride membrane using a semi-dry apparatus. The blots were blocked by incubation for 1 h at room temperature (RT) in TBS-T (20 mM Tris-HCl (pH 7.4), 150 mM NaCl, and 0.1% Tween 20) containing 5% skimmed milk or 3% bovine serum albumin. Subsequently, the blots were incubated with the primary antibodies overnight at 4 °C. After washing three times with TBS-T, the blots were incubated with the secondary antibodies for 1 h at RT. After another three rounds of washing with TBS-T, the blots were reacted with Immobilon Western Chemiluminescent HRP substrate, and the signals were detected using a LuminoShot 400Jr instrument (TAKARA Bio, Shiga, Japan). Intensity analysis was performed using ImageJ/Fiji (ver. 2.0) software [61].

### Immunostaining

Cells that were plated on glass-bottom dishes (MatTek, Ashland, MA, USA) were incubated with 10 nM CTB conjugated to Alexa488 for 30 min on ice, fixed with 4% paraformaldehyde (Sigma) for 15 min at RT, and then permeabilized with PBS containing 0.1% Triton X-100 for 5 min at RT. The cells were then washed three times with PBS, blocked with 0.1% bovine serum albumin in PBS for 1 h at RT, and incubated for 1 h at RT with the primary antibodies in the dark. After three washes (5 min each) with PBS, the cells were incubated with the secondary antibodies for 1 h at RT in the dark. After one wash with PBS-T and three washes (5 min each) with PBS, the cells were mounted in 75% glycerol. Images of the stained cells were acquired using a laser-scanning confocal microscope (LSM780, Carl Zeiss, Germany) equipped with a Plan-Apochromat × 63 immersion lens.

### Statistical analysis

Results were derived from experiments performed at least three separate times. The data are expressed as the mean ± SEM. Statistical analyses of the data were performed using two-tailed, unpaired Student’s *t*-tests. Homoscedasticity of the data was assumed (Microsoft Excel, Redmond, WA, USA) and *p* < 0.05 was considered statistically significant.

## Additional files

Additional file 1: **Figure S1.** Gray scale images of the localization of Flot2 and the other molecules before neural differentiation. (A–C) Undifferentiated P19C6 cells were fixed and stained with an anti-Flot2 antibody (b), Alexa488-conjugated Cholera toxin subunit B (a), an anti-Na/K ATPase α1 subunit antibody (NKA, A-c), an anti-Fyn antibody (B-c), and an anti-c-Src antibody (C-c), and their merged images are shown (e). In these merged images, the staining presented in a, b, and c is shown in blue, green, and red, respectively. Differential interference contrast images were also obtained (d). Scale bar shows 10 μm. Abbreviations: Flot2: Flotillin2; NKA: Na/K ATPase; CTB: Cholera toxin subunit B; DIC: Differential interference contrast. (PDF 292 kb)

Additional file 2: **Figure S2.** Gray scale images of the localization of Flot2 and the other molecules after neural differentiation. (A–C) Neural differentiated P19C6 cells were fixed and stained with an anti-Flot2 antibody (b), Alexa488-conjugated Cholera toxin subunit B (a), an anti-Na/K ATPase α1 subunit antibody (NKA, A-c), an anti-Fyn antibody (B-c), and an anti-c-Src antibody (C-c), and their merged images are shown (e). In these merged images, the staining presented in a, b, and c is shown in blue, green, and red, respectively. Differential interference contrast images were also obtained (d). Scale bar shows 20 μm. (D–E) Higher magnification images of the white boxes in A-e and C-e (blue: Cholera toxin subunit B; green: Flot2; red: NKA or c-Src). Scale bar shows 5 μm. The arrowheads indicate colocalized signals Flot2 and c-Src (E-b and E-c). Abbreviations: Flot2: Flotillin2; NKA: Na/K ATPase; CTB: Cholera toxin subunit B; DIC: Differential interference contrast. (PDF 318 kb)

## Data Availability

No applicable.

## Abbreviations

CTB: Cholera toxin subunit B
DRM: Detergent-resistant membrane
Flot1: Flotillin-1
Flot2: Flotillin-2
Frs: fractions
hMSCs: human multipotent stromal cells
MAP2: microtubule-associated protein 2
Neu: neural differentiated cells
NKA: Na/K ATPase
Oct-3: Octamer transcription factor-3
UD: undifferentiated cells
WB: Western blotting
