# Supplementary figures and images for: The Flot2 component of the lipid raft changes localization during neural differentiation of P19C6 cells
Source: BMC Mol Cell Biol. 2019 Aug 27;20:38. doi: 10.1186/s12860-019-0225-0 (PMC6712619; doi:10.1186/s12860-019-0225-0)

**A**

GM1

Flot2

NKA

DIC

merge

**a****b****c****d****e****B**

GM1

Flot2

Fyn

DIC

merge

**a****b****c****d****e****C**

GM1

Flot2

c-Src

DIC

merge

**a****b****c****d****e**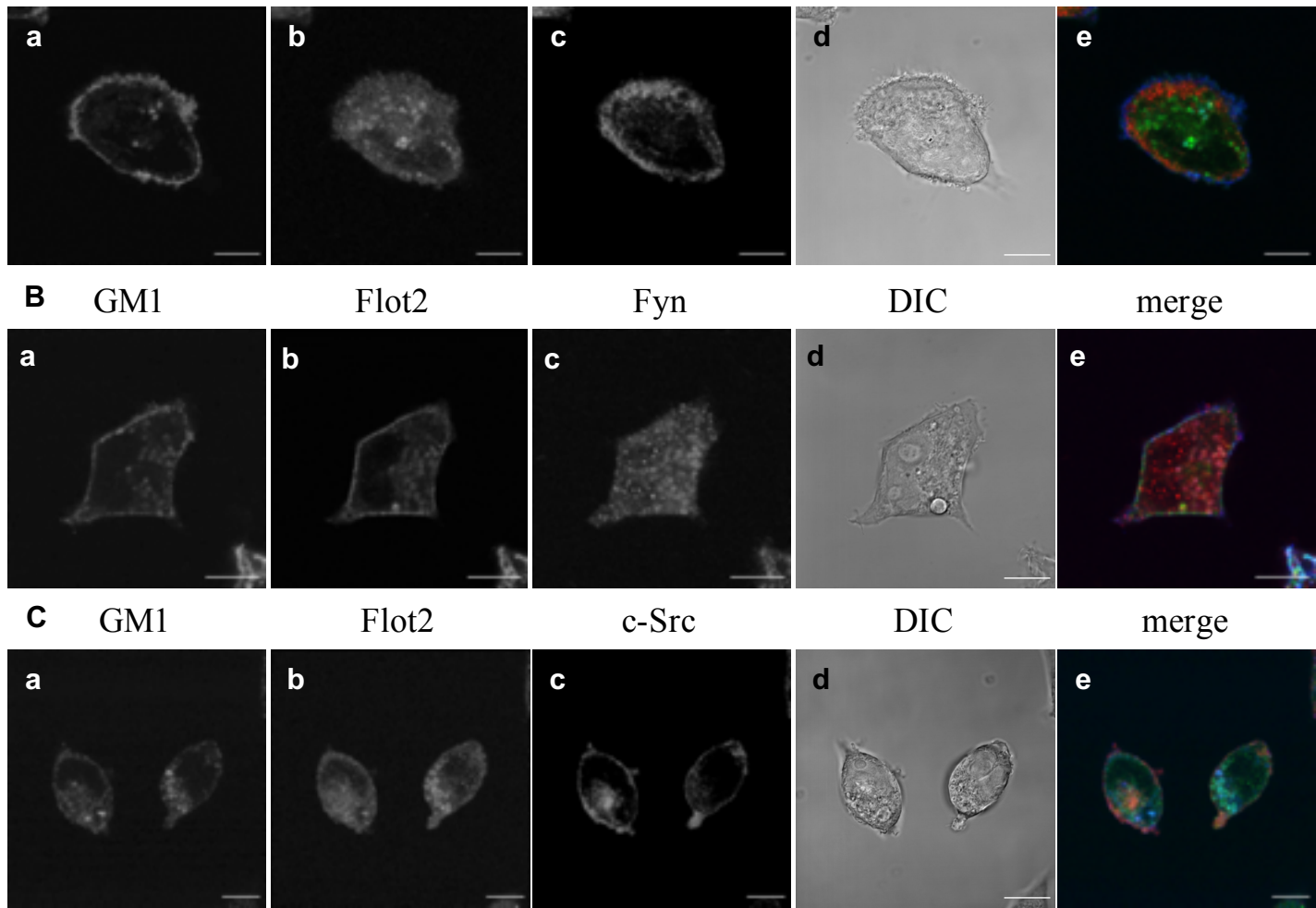

Supplement: Supplementary file 1 — Figure S1. Gray scale images of the localization of Flot2 and the other molecules before neural differentiation. (A–C) Undifferentiated P19C6 cells were fixed and stained with an anti-Flot2 antibody (b), Alexa488-conjugated Cholera toxin subunit B (a), an anti-Na/K ATPase α1 subunit antibody (NKA, A-c), an anti-Fyn antibody (B-c), and an anti-c-Src antibody (C-c), and their merged images are shown (e). In these merged images, the staining presented in a, b, and c is shown in blue, green, and red, respectively. Differential interference contrast images were also obtained (d). Scale bar shows 10 μm. Abbreviations: Flot2: Flotillin2; NKA: Na/K ATPase; CTB: Cholera toxin subunit B; DIC: Differential interference contrast. (PDF 292 kb) [file 12860_2019_225_MOESM1_ESM.pdf]

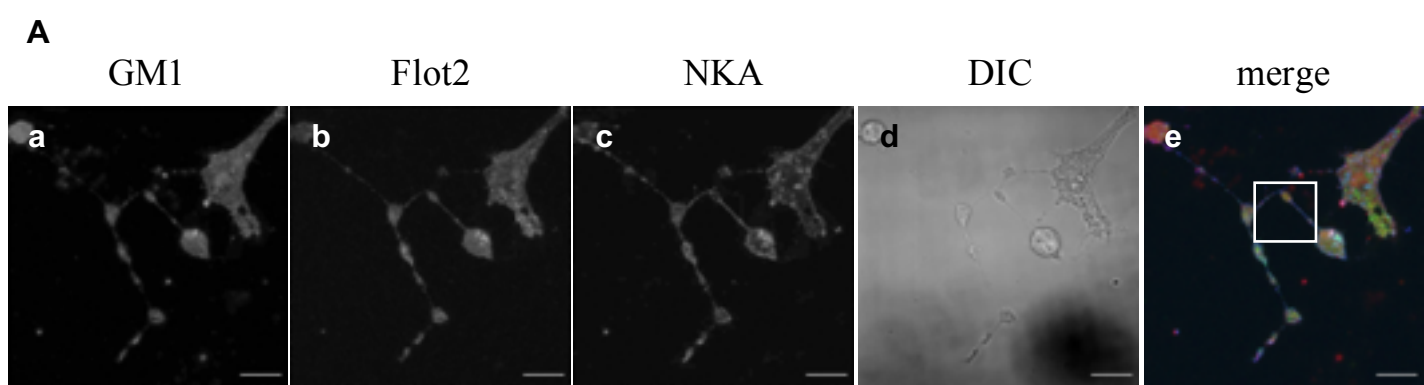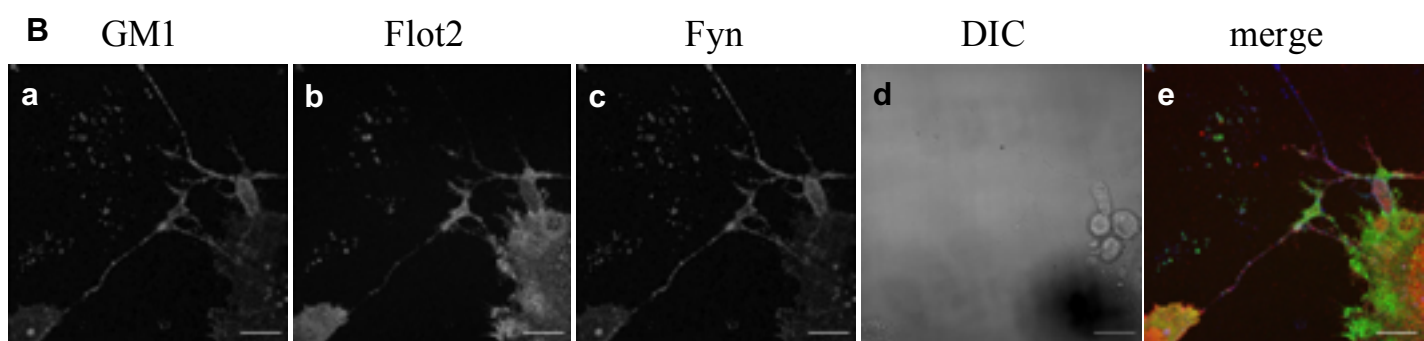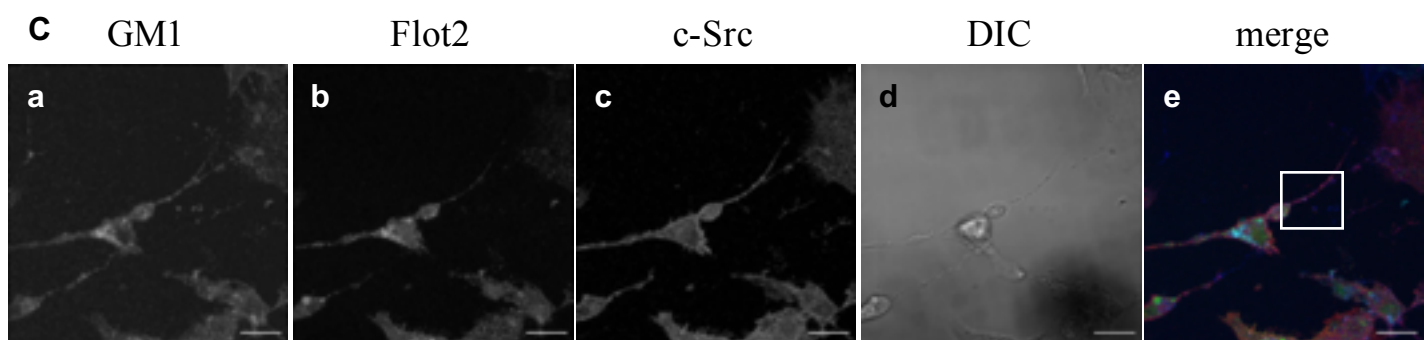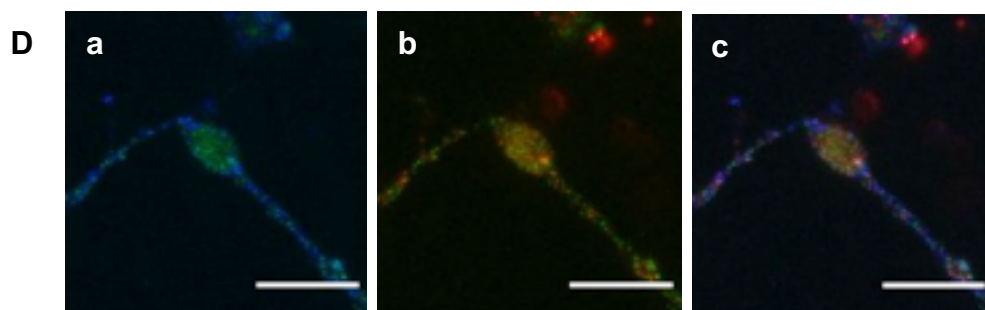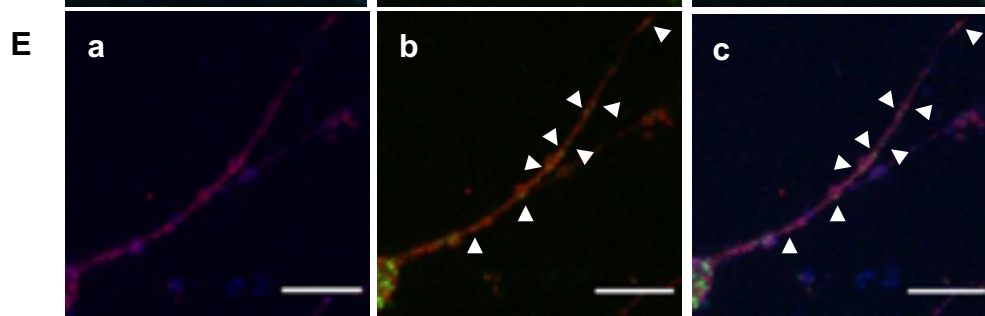

Supplement: Supplementary file 2 — Figure S2. Gray scale images of the localization of Flot2 and the other molecules after neural differentiation. (A–C) Neural differentiated P19C6 cells were fixed and stained with an anti-Flot2 antibody (b), Alexa488-conjugated Cholera toxin subunit B (a), an anti-Na/K ATPase α1 subunit antibody (NKA, A-c), an anti-Fyn antibody (B-c), and an anti-c-Src antibody (C-c), and their merged images are shown (e). In these merged images, the staining presented in a, b, and c is shown in blue, green, and red, respectively. Differential interference contrast images were also obtained (d). Scale bar shows 20 μm. (D–E) Higher magnification images of the white boxes in A-e and C-e (blue: Cholera toxin subunit B; green: Flot2; red: NKA or c-Src). Scale bar shows 5 μm. The arrowheads indicate colocalized signals Flot2 and c-Src (E-b and E-c). Abbreviations: Flot2: Flotillin2; NKA: Na/K ATPase; CTB: Cholera toxin subunit B; DIC: Differential interference contrast. (PDF 318 kb) [file 12860_2019_225_MOESM2_ESM.pdf]
